# RNA-sequencing expression profile and functional analysis of retinal pigment epithelium in atrophic age-related macular degeneration

**DOI:** 10.7555/JBR.37.20230320

**Published:** 2024-05-29

**Authors:** Miao Xu, Yan Gao, Wenjie Yin, Qinghuai Liu, Songtao Yuan

**Affiliations:** Department of Ophthalmology, the First Affiliated Hospital of Nanjing Medical University, Nanjing, Jiangsu 210029, China

**Keywords:** age-related macular degeneration, retinal pigment epithelium, high-throughput RNA-sequencing, bioinformatics analysis

## Abstract

The retinal pigment epithelium (RPE) is fundamental to sustaining retinal homeostasis. RPE abnormality leads to visual defects and blindness, including age-related macular degeneration (AMD). Although breakthroughs have been made in the treatment of neovascular AMD, effective intervention for atrophic AMD is largely absent. The adequate knowledge of RPE pathology is hindered by a lack of the patients' RPE datasets, especially at the single-cell resolution. In the current study, we delved into a large-scale single-cell resource of AMD donors, in which RPE cells were occupied in a substantial proportion. Bulk RNA-seq datasets of atrophic AMD were integrated to extract molecular characteristics of RPE in the pathogenesis of atrophic AMD. Both *in vivo* and *in vitro* models revealed that carboxypeptidase X, M14 family member 2 (CPXM2), was specifically expressed in the RPE cells of atrophic AMD, which might be induced by oxidative stress and involved in the epithelial-mesenchymal transition of RPE cells. Additionally, silencing of *CPXM2* inhibited the mesenchymal phenotype of RPE cells in an oxidative stress cell model. Thus, our results demonstrated that CPXM2 played a crucial role in regulating atrophic AMD and might serve as a potential therapeutic target for atrophic AMD.

## Introduction

Age-related macular degeneration (AMD) is the most common cause of severe visual acuity loss among the elderly population in developed countries ^[ 1] ^. AMD is classified into atrophic AMD and neovascular AMD ^[ 2] ^. Atrophic AMD, considered the terminal stage of the condition, leads to a progressive central visual field loss accompanied by the atrophy of photoreceptor, retinal pigment epithelium (RPE), and choriocapillaris ^[ 3] ^. While anti-angiogenesis therapies are effective for treating neovascular AMD, there are limited approaches available to deal with atrophic AMD.

RPE is essential for normal vision maintenance possessing barrier and phagocytic function, whose dysfunction may lead to retinal degenerative diseases, such as AMD ^[ 4] ^. Sustained intracellular oxidative stress, chronic inflammation, genetic susceptibility, and environmental factors are the proposed mechanisms for RPE abnormalities in atrophic AMD ^[ 5] ^. Currently, abnormal complement activation has been verified to be involved in RPE inflammatory phenotype shift and cell dysfunction caused by immune cell recruitment in atrophic AMD ^[ 6] ^. Two complement inhibitors, including eculizumab and zimura, have been approved by the U.S. Food and Drug Administration (FDA) in the atrophic AMD therapy ^[ 7– 8] ^. Concerning the elevated intracellular oxidative stress, anti-oxidative injury complements, including vitamin C and E, zinc, and lutein, have been confirmed to reduce the risk of neovascular AMD development within five years, but not in atrophic AMD ^[ 9] ^. Thus, identifying novel targets for anti-oxidative stress is imperative in the development of effective treatments for atrophic AMD.

RPE-based therapeutics offer the promise of a long-term substitute for atrophic tissue, emphasizing that the degeneration of RPE is the initial step for atrophic AMD ^[ 10] ^. Oxidative stress along with epithelial-mesenchymal transition (EMT) is known to contribute to RPE dysfunction ^[ 11] ^. The retinal unique anatomical and metabolic characteristics make it susceptible to the reactive oxygen species (ROS) that are further increased by environmental exposure such as smoking ^[ 12] ^. In addition, genetic variants in genes related to oxidative stress multiply the risk of AMD, further evidencing the effects of ROS on the pathogenesis of AMD ^[ 12] ^. EMT serves as an impetus in subretinal fibrosis, the terminal stage of AMD, by inducing mesenchymal cells that produce extracellular matrix ^[ 13] ^. In subretinal fibrotic lesions, mesenchymal cells that mainly generate matrix may be partly derived from RPE cells undergoing EMT ^[ 13] ^. RPE cells of patients with atrophic AMD expressed more mesenchymal markers and less E-cadherin, indicating an EMT involvement ^[ 14] ^.

It is difficult to identify subsets of disease-specific cells using traditional microarray and RNA-seq techniques ^[ 15] ^. Single-cell RNA sequencing (scRNA-seq) offers a solution, allowing for the high-resolution and in-depth study of transcriptomes in each cell subpopulation within highly heterogeneous samples, thus overcoming these limitations ^[ 16] ^. Additionally, single-nucleus RNA-sequencing (snRNA-seq), instead of scRNA-seq, is more appropriate for non-fresh tissue samples, such as human choroid tissues, which extends the collection time of human samples and minimizes the dissociation bias ^[ 17– 18] ^. To date, investigations for AMD at the single-cell resolution showed a preference for the transcriptomic characteristics of choriocapillaris ^[ 19] ^, partly because of the paucity of RPE cells. Recently, Orozco *et al* ^[ 20]^ provided a large-scale and multi-omics dataset derived from well-characterized donor eyes, including plenty of RPE cells from single-nucleus transcriptome. They comprehensively elucidated underlying factors driving different stages of AMD, but lacked cell-specific expression patterns from the phenotyped donors.

**Figure 1:**
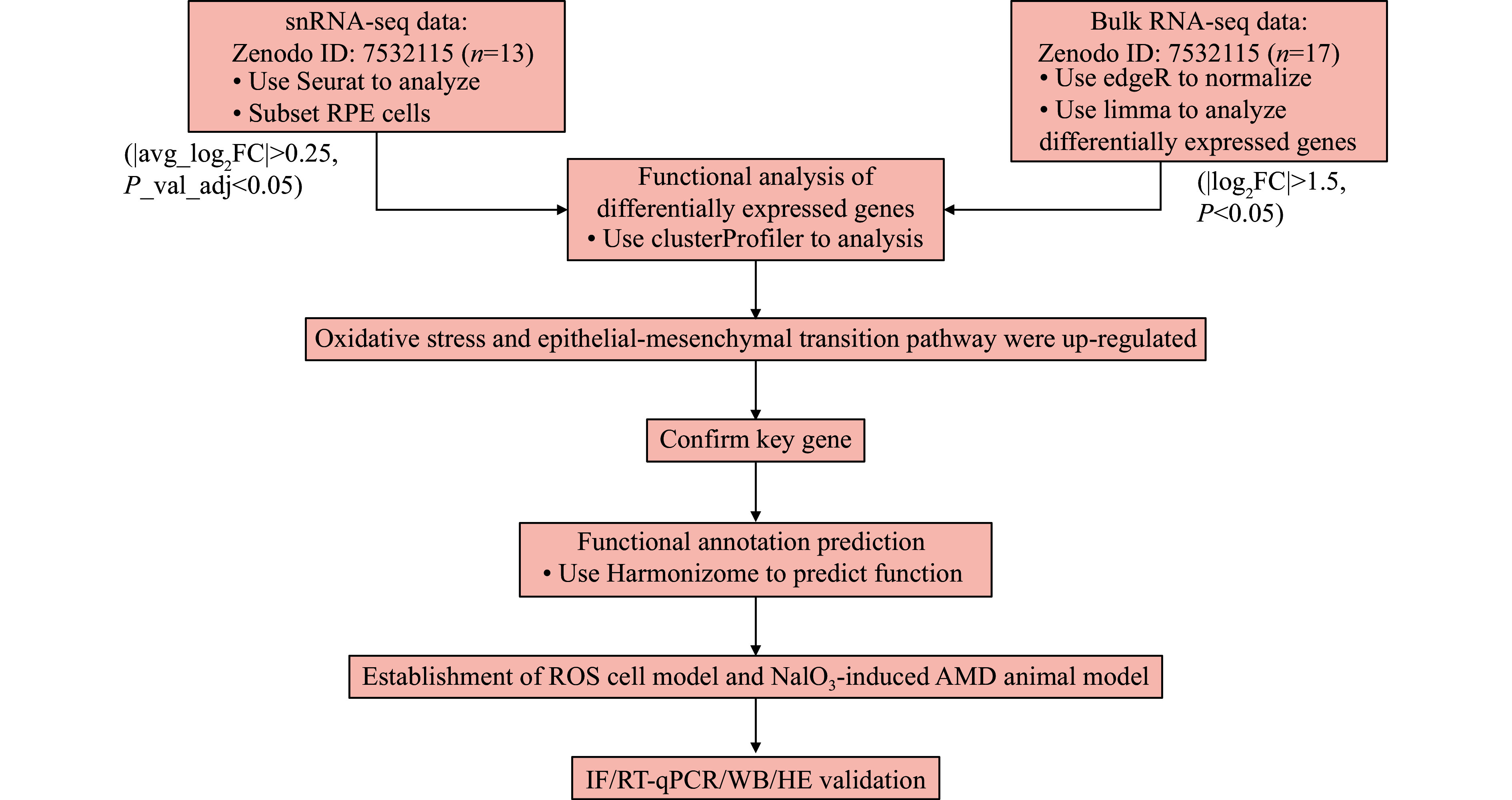
The flowchart for joint analysis of snRNA-seq and bulk RNA-seq data.

In the current study, we conducted a conjoint analysis of snRNA-seq and bulk RNA-seq of AMD patients from the Zenodo database to identify the key genes that may contribute to the EMT of RPE cells, followed by a validation of the role of these genes in a hydrogen peroxide (H _2_O _2_)-induced cell model *in vitro* and a sodium iodate (NaIO _3_)-induced atrophic AMD mouse model *in vivo.* The flow chart of the current study is shown in ***Fig. 1***.

## Materials and methods

### Data collection and preprocessing

We obtained 13 snRNA-seq data (six from AMD groups and seven from controls) of human AMD and 17 bulk RNA-seq data (nine from geographic atrophy groups and eight from controls) of human atrophic AMD (ID No. 7532115, https://zenodo.org/records/7532115) from the Zenodo database. Zenodo is an open science platform that hosts and references research data and software from different disciplines and sources. High-throughput sequencing results of snRNA-seq and bulk RNA-seq were completed by the Illumina HiSeq 4000 platform ^[ 20] ^. The socio-demographic information corresponding to the data used in the current study is shown in ***Supplementary Table 1*** (available online).

### Analysis of sequencing data from the Zenodo database

We first integrated snRNA-seq data from AMD patients using the Seurat software package that was specifically designed for quality control, analysis, and exploration of single-cell RNA-seq data ^[ 21] ^. The data were visualized by the Uniform Manifold Approximation and Projection (UMAP) method based on dimension reduction, and then the cell types were annotated using the accepted markers. The differentially expressed genes (DEGs) in RPE cells were identified using the FindMarkers function. The threshold was chosen as the |log _2_(fold change)| ≥ 0.25 and the *P*-value of Benjamin's correction < 0.05. We subsequently performed a principal component analysis (PCA) to examine the sample heterogeneity of bulk RNA-seq data. Package edgeR was used to identify DEGs in bulk RNA-seq data, where the threshold was selected as an |log _2_(fold change)| ≥ 1.5 and a *P*-value < 0.05.

### Gene Ontology (GO) enrichment analysis

In GO enrichment analysis, genes were clustered according to their functions to provide insights into differential gene expression. The analysis of gene enrichment was performed using the enrichGO functions in the R package clusterProfiler ^[ 22] ^. *P* < 0.05 was used as the threshold for defining significantly enriched GO terms.

### Cell culture and treatments

RPE cell line ARPE-19 was obtained from American Type Culture Collection (Manassas, VA, USA) and grown in Dulbecco's Modified Eagle's/F 12 Medium (DMEM; Gibco, Grand Island, NY, USA), supplemented with 10% fetal bovine serum (FBS; SenBeiJia, Nanjing, China) and 1% penicillin-streptomycin (Gibco). Cells were cultured at 37 ℃ in a humidified 5% CO _2_ and 21% oxygen incubator. For immunofluorescence staining, 1 × 10 ^5^ cells were seeded on the coverslips in each of the 24-well plates. For quantitative reverse transcription PCR (qRT-PCR) and Western blotting analyses, 2.5 × 10 ^5^ cells were seeded in each of the six-well plates. After seeding, ARPE-19 cells were cultured in a complete medium for 24 h, and when the level of cell confluency reached about 80%, they were treated with H _2_O _2_ or transfected with small interfering RNA (siRNA). These cells were incubated with 200 mol/L H _2_O _2_ (Sigma, St. Louis, MO, USA) for 4 h and then converted to a complete medium, and they were harvested 24 h after transferring to a complete medium.

### Establishment of the atrophic AMD mouse model

The current study used animals according to recommendations of the Association for Research in Vision and Ophthalmology (ARVO) and was approved by the Institutional Review Board of Nanjing Medical University (Approval No. IACUC-1902009). The establishment of an atrophic AMD mouse model with photoreceptor impairment was performed as previously described ^[ 23] ^. Six-week-old male C57BL/6J mice were divided into two groups, including saline-injected control mice ( *n* = 4) and NaIO _3_-injected mice ( *n* = 6). In brief, C57B6L/J mice underwent a tail vein injection of 35 mg/kg sterile 1% NaIO _3_ in saline. The control mice were injected with the respective volume of 0.9% NaCl (Otsuka Pharma, China; 100 μL) *via* the tail vein. Only male mice were used in this experiment, so that no littermates were produced. The mice were kept on a standard 12-h light/12-h dark cycle. The eyes were enucleated 14 days after treatment, and the mice were anesthetized using 2% isoflurane flowing through a face mask before each surgical and experimental procedure.

### Immunofluorescence staining

The eyes of C57BL/6 mice were harvested and fixed in FAS eyeball fixative (Servicebio, Wuhan, China) for 1 h at room temperature, placed in 10% and 20% sucrose solution for 10 min, respectively, and finally in 30% sucrose recipe at 4 ℃ overnight. Afterward, the eyes were embedded in O.C.T. Compound (Tissue-Tek, Naperville, IL, USA) and sliced into 8-μm sections before being stored at −80 ℃. The collected eyes were placed in a vial containing phosphate-buffered saline (PBS, Gibco) on ice. The corneas were held with forceps, and a small incision was made in the cornea using scissors. The anterior segment, including the cornea, sclera, and lens, was then removed. Cryosections were washed with PBS for 5 min each time, then blocked with 10% FBS, 2% serum, and 0.2% Triton X-100 for 1 h at room temperature. The sections were subsequently incubated with the antibodies against 4-hydroxynonenal (4-HNE, 1∶200; Cat. #bs-6313R, Bioss, Beijing, China), CPXM2 (1∶200; Cat. #DF9346, Affinity Biosciences, Cincinnati, OH, USA), SNAIL (1∶200; Cat. #ab180714, Abcam, Cambridge, UK), fibronectin 1 (FN1, 1∶200; Cat. #YM3137, Immunoway, Beijing, China), α-smooth muscle actin (αSMA, 1∶200; Cat. #ab124964, Abcam), retinal pigment epithelium 65 kDa protein (RPE65, 1∶300; Cat. #ab231782, Abcam), or rhodopsin (1∶200; Cat. #ab98887, Abcam) at 4 ℃ overnight. Fluorescence-conjugated anti-rabbit secondary antibodies (1∶1000; Cat. #ab175692, Abcam) and anti-mouse (1∶1000; Cat. #ab150111, Abcam) were incubated at room temperature for 1 h. The nuclei of the cells were stained using DAPI (Sigma-Aldrich). Fluorescence was observed with a Leica microscope.

### qRT-PCR analysis

qRT-PCR was performed to verify the levels of mRNAs in cells treated with *CPXM2* siRNA. The qRT-PCR was carried out according to the previously described instructions ^[ 24] ^. Total RNA was isolated according to the above description. PrimeScript RT kit (Takara, Otsu, Shiga, Japan) was used to reverse transcriptase RNA, and qRT-PCR was performed on the Step One Plus system (Applied Biosystems, Waltham, MA, USA). *ACTB* was used as an internal reference for mRNAs. Primer sequences used for qRT-PCR are shown in ***Supplementary Table 2*** (available online).

### Retinal morphometric analysis

An analysis of morphometric parameters was carried out using hematoxylin and eosin (H&E, Cat. #C0105S; Beyotime, Shanghai, China)-stained cross-sections of murine eyes. To evaluate the number of photoreceptor cells, the nuclei were manually counted every 250 µm along longitudinal stretches of 60 µm, using ImageJ software.

### Western blotting

ARPE-19 cells were harvested and dissociated in a RIPA lysis buffer (Beyotime). Protein samples were separated by 10% SDS-PAGE, and then transferred to polyvinylidene difluoride membranes (Millipore, Billerica, MA, USA). The blots were subsequently incubated with antibodies against 4-HNE (1∶200; Cat. #bs-6313R, Bioss), CPXM2 (1∶200; Cat. #DF9346, Affinity Biosciences), FN1 (1∶200; Cat. #YM3137, Immunoway), αSMA (1∶200; Cat. #ab124964, Abcam), SNAIL (1∶200, Cat. #ab180714, Abcam), zona occludens-1 (ZO-1, 1∶200; Cat. #21773-1-AP, Proteintech, Wuhan, China), bestrophin-1 (BEST1, 1∶200; Cat. #YN5662, Immunoway) or β-actin (1∶200; Cat. #81115-1-RR, Proteintech). After an overnight incubation at 4 ℃, the blots were incubated with HRP-conjugated secondary anti-rabbit (1∶1000; Cat. #A0208, Beyotime) or anti-mouse (1∶1000; Cat. #A0216, Beyotime) antibodies. To detect specific signals, the protein bands were detected using an enhanced chemiluminescence detection kit (Vazyme, Nanjing, China). The membrane was visualized using a chemiluminescence detection system (Tanon, Shanghai, China) and band intensities were analyzed using ImageJ software.

### Transfection with siRNA

As prescribed by the manufacturer, *CPXM2* siRNA (Ribobio, Guangzhou, China) was transfected into ARPE-19 cells at 80% confluence with the riboFECTTM CP transfection kit (Ribobio). On a six-well plate, 120 μL Opti-MEM medium consisting of 12 μL riboFECT CP was premixed with 100 nmol/L *CPXM2* siRNA or 100 nmol/L negative control, respectively. A qRT-PCR analysis was performed after the cells were transduced for 48 h to demonstrate silence efficiency. The sequence of siRNA targeting *CPXM2*: 5′-GCCGAAGGTTTCACTGCAT-3′.

### Statistical analysis

At least three independent experiments were performed for each analysis. Except where otherwise noted, data were presented as means with standard errors of the mean. The statistical analysis was conducted using the GraphPad Prism software (Version 9.5.0; GraphPad Inc., San Diego, CA, USA). The Shampiro-Wilk test was used to confirm the distribution's normality. Statistical comparisons were made using the two-tailed unpaired Student's *t*-test or an analysis of variance (ANOVA) followed by Tukey's Honestly-Significant Difference post hoc test when appropriate. We calculated two-sided *P*-values and designated the significance with *P* < 0.05.

## Results

### Integration of snRNA-seq datasets and RPE cell reanalysis

**Figure 2:**
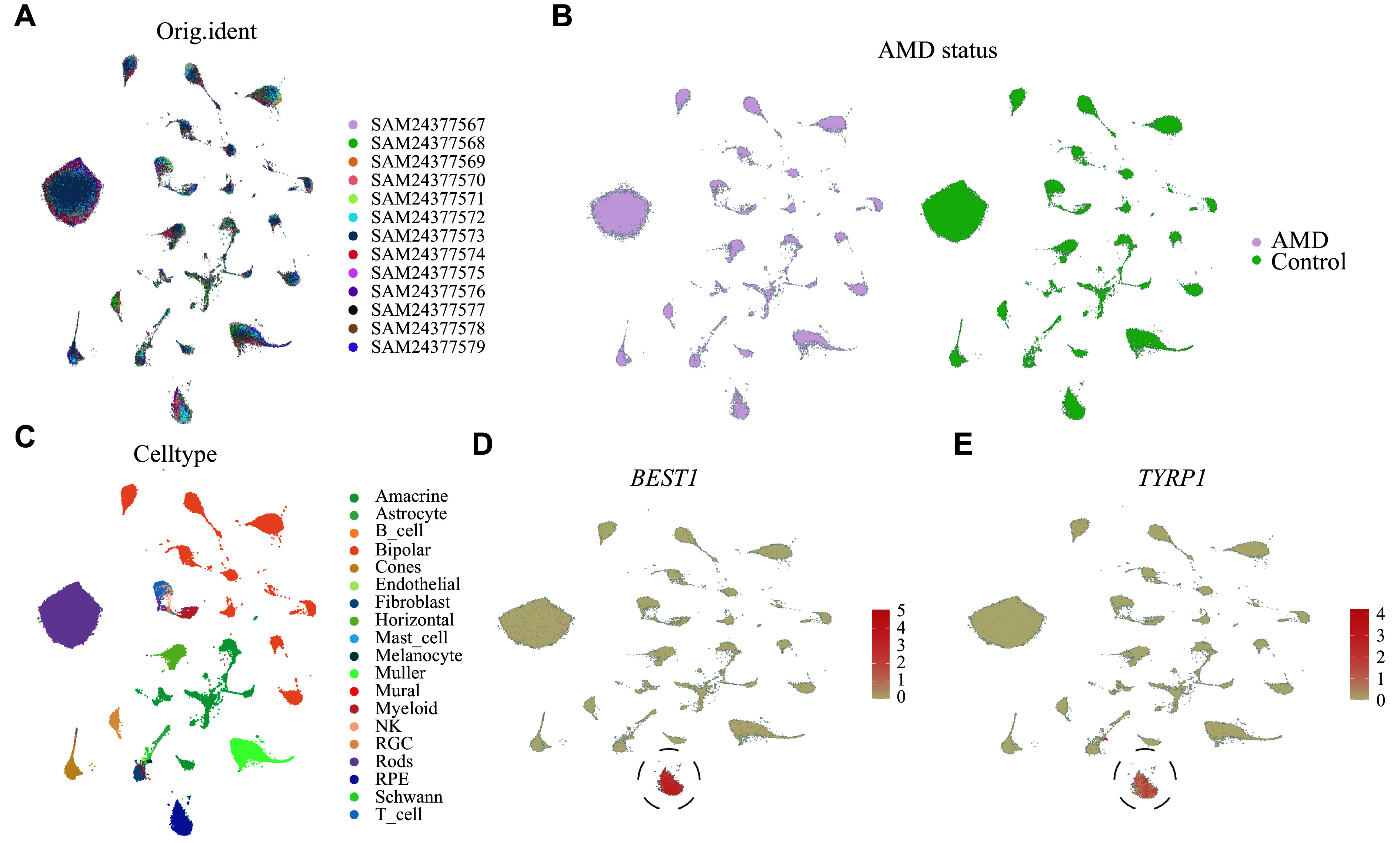
Single-nucleus RNA-seq atlas of the retina, choroid, and RPE tissues from AMD and healthy donors.

To determine the signature of RPE cells in atrophic AMD, we analyzed the snRNA-seq dataset that comprised seven healthy individuals and six patients with advanced AMD. Quality control methods were used to remove any outliers ( ***Supplementary Fig. 1A***– ***1C***, available online). Data consolidation was performed using the IntegrateData function. After integration, no noticeable batch effect was identified between samples and disease groups ( ***Fig. 2A*** and ***2B***). For accurate classification of these cell types, known candidate features for cell identification were employed ( ***Fig. 2C*** and ***Supplementary Fig. 1D*** [available online]). RPE cells were specifically targeted based on their gene expression signatures, which included *BEST1* and tyrosinase-related protein 1 ( *TYRP1*) ( ***Fig. 2D*** and ***2E***).

**Figure 3:**
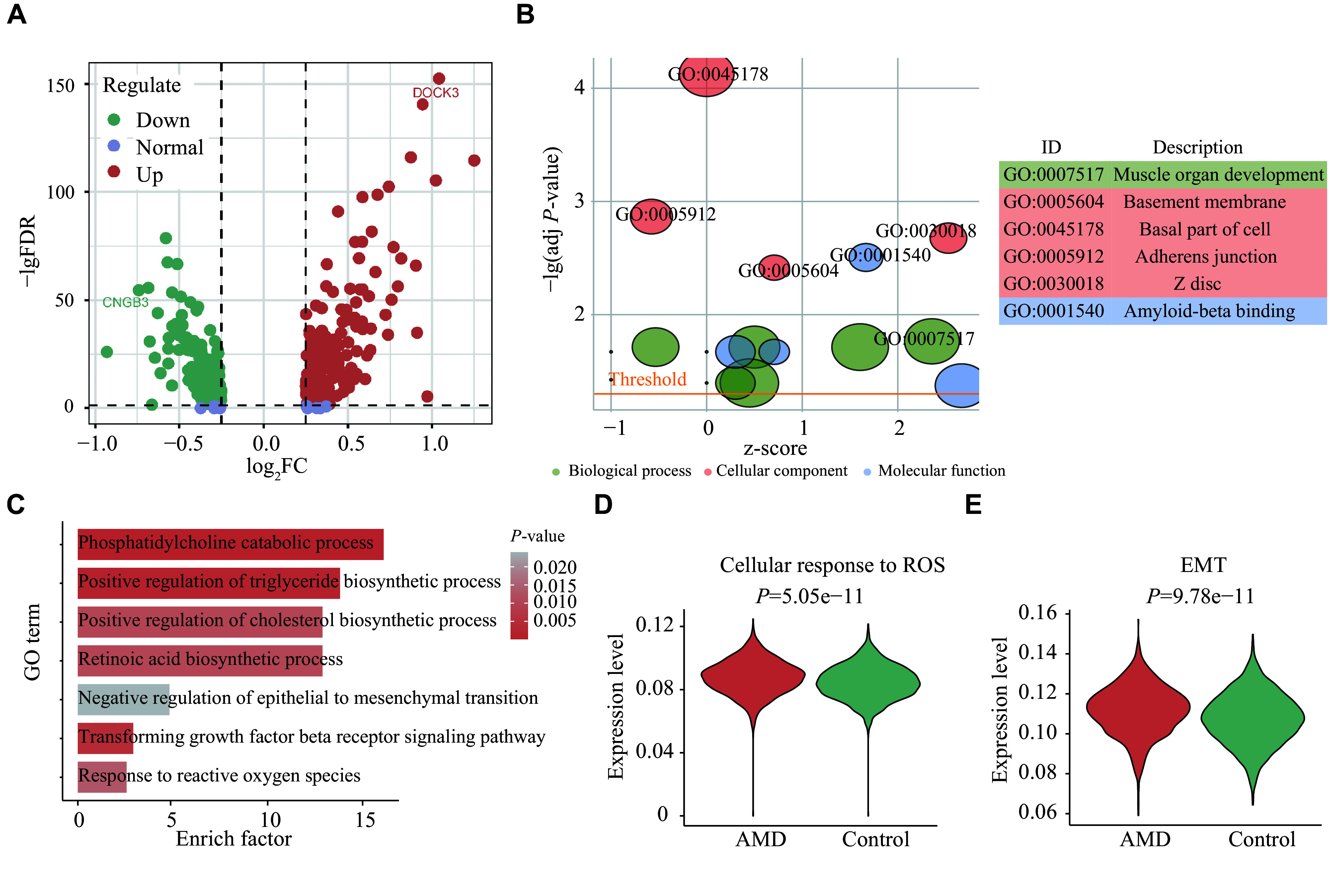
Single-cell analysis of RPE cells from AMD and healthy donors.

To investigate the underlying pathological mechanisms associated with RPE cell abnormalities, we performed a re-analysis of the RPE cells. DEGs within RPE cells are presented in a dot plot ( ***Fig. 3A ***) and the top 50 DEGs are listed in ***Supplementary Table 3*** (available online). Furthermore, we compared our findings with the disease-specific genes of RPE cells obtained through pseudobulk analysis by Orozco *et al* ^[ 20] ^, revealing a partial overlap with our results ( ***Supplementary Table 4 ***, available online). GO enrichment analysis was applied to ascertain dysregulated biological processes, cellular components, and molecular functions within AMD samples ( ***Fig. 3B ***). The amyloid-beta (Aβ) binding pathway showed specific enrichment ( ***Fig. 3B ***), consistent with previous studies reporting Aβ deposition in RPE cells, which is believed to be associated with AMD progression ^[ 25] ^, further supporting the abnormal state of RPE in AMD. Additionally, Aβ production and accumulation were correlated with abnormal content and distribution of cholesterol in the membrane ( ***Fig. 3C*** and ***Supplementary Table 5*** [available online]). Moreover, oxidative stress and EMT pathways were found to be activated in AMDRPE cells ( ***Fig. 3D*** and ***3E ***).

### Analysis of bulk RNA-seq datasets from atrophic AMD patients

**Figure 4:**
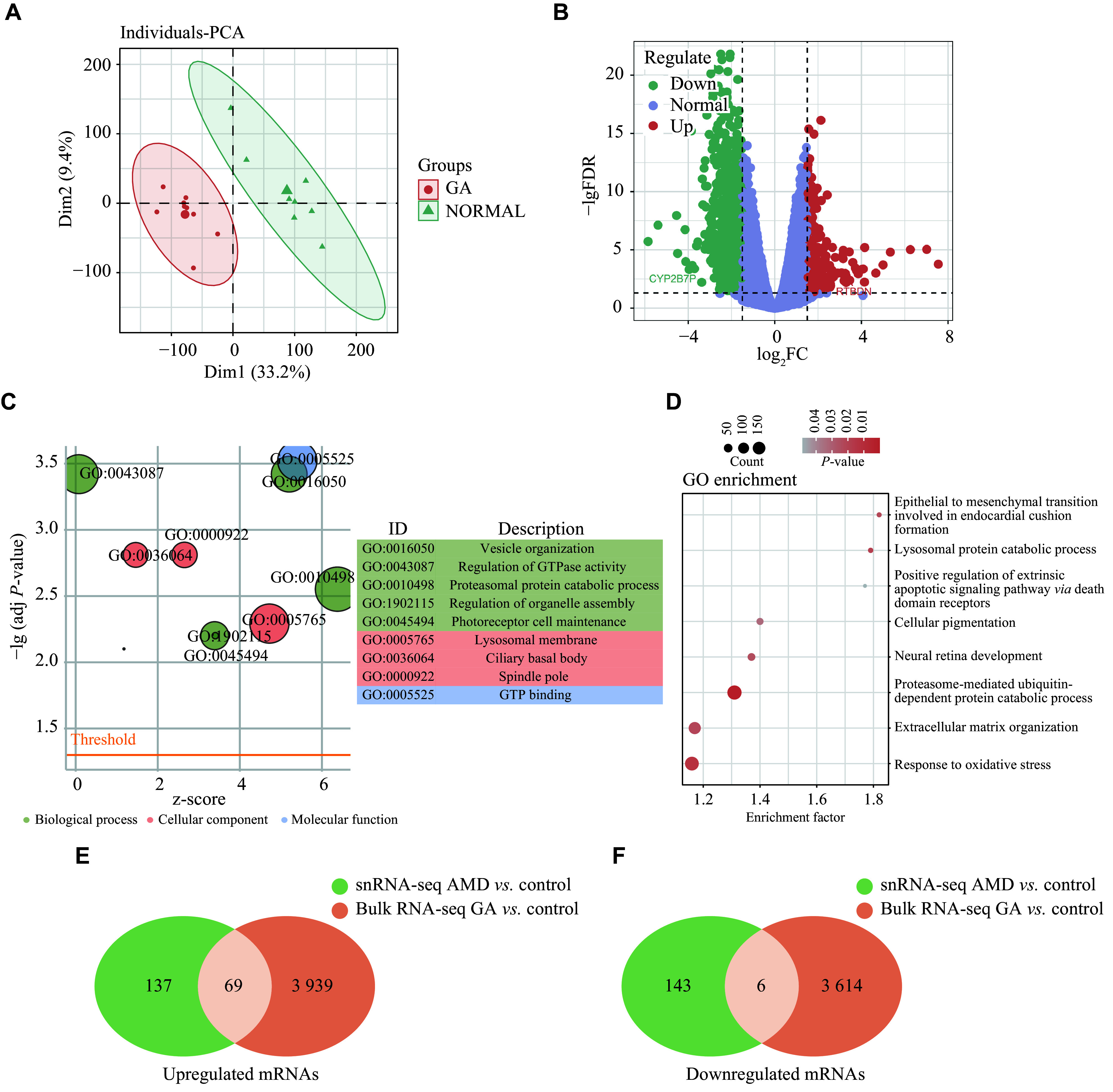
Bulk RNA-seq analysis of RPE cells from atrophic AMD and healthy donors.

To investigate the underlying pathogenesis in RPE cells of atrophic AMD, we analyzed bulk RNA-seq data derived from patients with geographic atrophy. Given that atrophic AMD primarily affects the macular RPE-choroidal complex, only macular tissue samples from atrophic AMD patients were included, with healthy samples used as controls. We performed PCA to evaluate the consistency of the samples, and observed a good intra-group consistency and significant differences between groups ( ***Fig. 4A ***). The DEGs in macular tissues were depicted in a volcano plot ( ***Fig. 4B ***), and the top 50 DEGs were listed in ***Supplementary Table 6*** (available online). GO enrichment analysis indicated the activation of numerous pathways associated with photoreceptor cells, consistent with the pathologic phenotype of atrophic AMD and severe photoreceptor damage ( ***Fig. 4C ***). Additionally, oxidative stress and EMT pathways were also found to be elevated in atrophic AMD ( ***Fig. 4D ***), and the top 50 significantly enriched GO terms of bulk RNA-seq from geographic atrophy samples were listed in ***Supplementary Table 7*** (available online). The observations reveal putative causal roles of these pathways in the disease tissues.

### Identification of pivotal RPE-specific genes involved in the development of atrophic AMD

**Figure 5:**
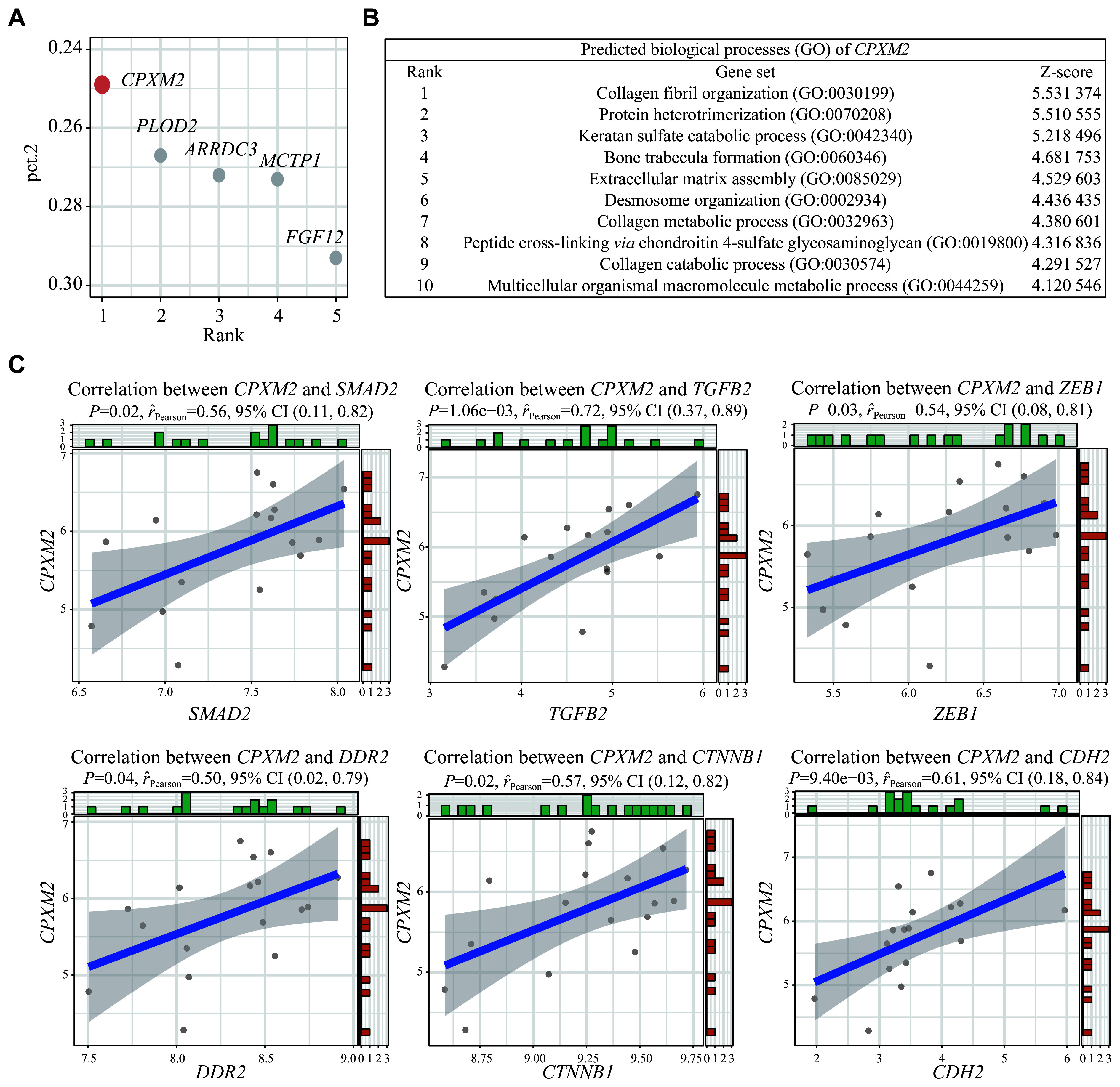
CPXM2, which is driven by oxidative stress, mediates the mesenchymal transition of RPE.

To elucidate the core regulatory genes implicated in atrophic AMD, we performed the Venn diagram analysis to identify the DEGs shared by both bulk and single-cell data. The results showed that 69 genes were upregulated, and six genes were downregulated in RPE cells from atrophic AMD samples, compared with those from the healthy controls ( ***Fig. 4E*** and ***4F***). Further screening was performed using the single-cell DEGs, with a criterion for specific expression of the gene in RPE cells (pct.2 < 0.3). The identified genes were ranked based on their specificity in a descending order, with the *CPXM2* gene ranking the first ( ***Fig. 5A***). We further used Harmonizome to predict the potential functions of *CPXM2*, and found that the top 10 functions were similar to those of mesenchymal cells, such as collagen fiber organization and extracellular matrix assembly, suggesting that CPXM2 may promote the depolarization of RPE ( ***Fig. 5B***). Importantly, the expression of *CPXM2* showed a significant positive correlation with several genes involved in EMT, such as *SMAD2*, *TGFB2*, and *ZEB1* ( ***Fig. 5C***). Based on these findings, we hypothesized that CPXM2 expression level might be influenced by oxidative stress and contribute to the EMT of RPE cells, ultimately leading to the development of atrophic AMD.

### Construction and validation of oxidative stress cell models

**Figure 6:**
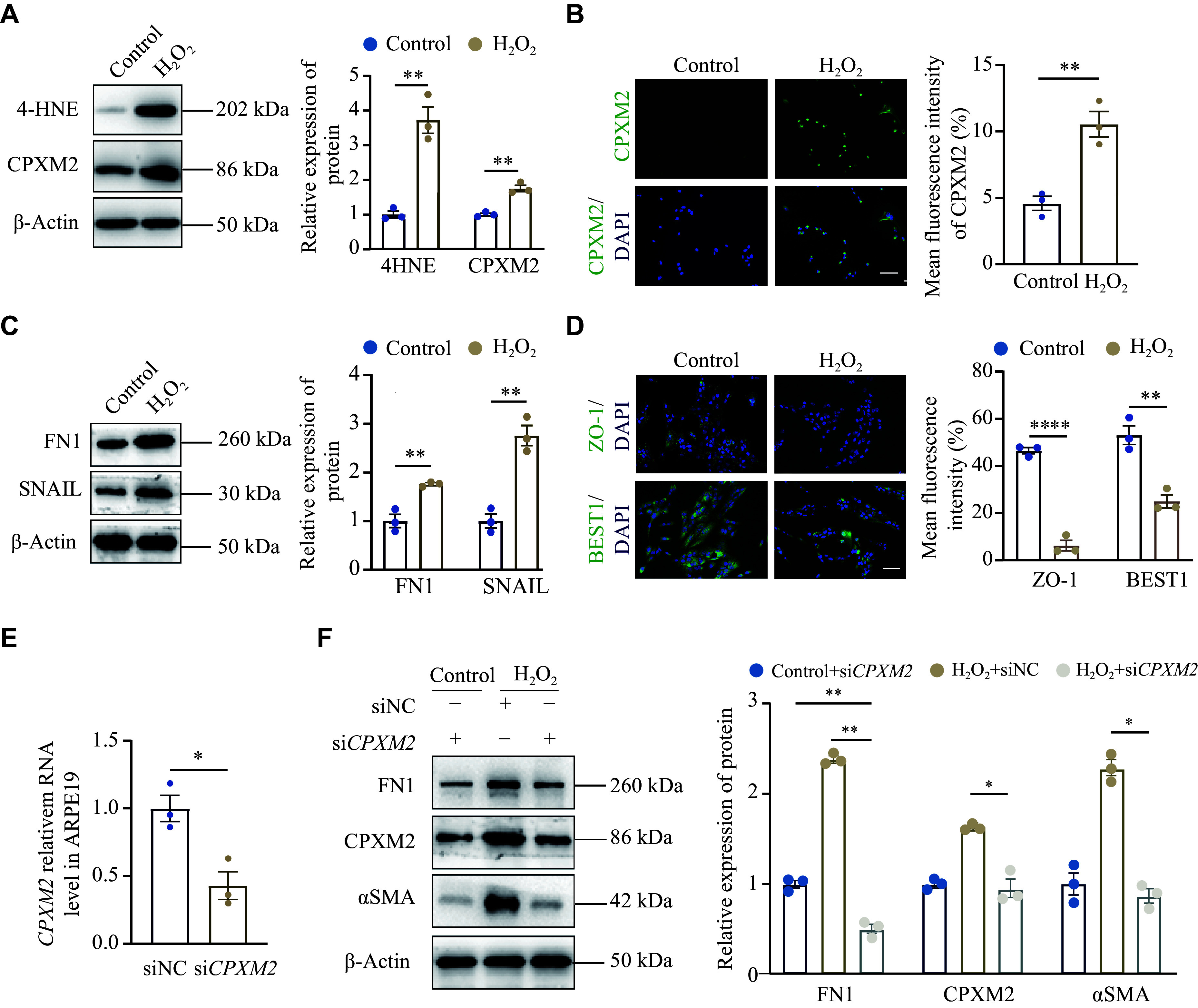
Validation of the ROS-CPXM2-EMT axis in oxidative stress cell models.

To validate the ROS-CPXM2-EMT axis, we established a cellular model of oxidative stress for further analysis. The level of 4-HNE, a product of lipid peroxidation and a potent indicator of oxidative damage ^[ 26] ^, was significantly elevated in the H _2_O _2_-treated RPE cells than in the control group ( ***Fig. 6A***), thereby confirming the successful establishment of the oxidative stress cell model. Simultaneously, the CPXM2 expression level was significantly increased along with oxidative stress response ( ***Fig. 6A*** and ***6B***). Additionally, the levels of EMT-related proteins, specifically FN1 and SNAIL ^[ 27] ^, were significantly increased in the H _2_O _2_-treated RPE cells ( ***Fig. 6C***). Furthermore, we found that the expression levels of ZO-1 (a major structural protein of tight junctions) and BEST1 (a Ca ^2+^-activated Cl ^−^ channel that mainly expressed in RPE cells) were significantly decreased in RPE cells after the H _2_O _2_ treatment ( ***Fig. 6D***). These results indicate that oxidative injury in RPE cells may contribute to the EMT and dysfunction of RPE cells, and that CPXM2 may participate in this process.

To investigate the role of CPXM2 in EMT, we knocked down the expression of *CPXM2* in ARPE-19 cells using siRNA targeting *CPXM2* ( ***Fig. 6E***). Both the mRNA and protein expression levels of αSMA and FN1 were significantly decreased upon the down-regulation of CPXM2 ( ***Fig. 6E*** and ***6F***), indicating that decreased CPXM2 expression effectively rescued the mesenchymal transition of RPE cells.

### Construction and validation of the atrophic AMD mouse model

**Figure 7:**
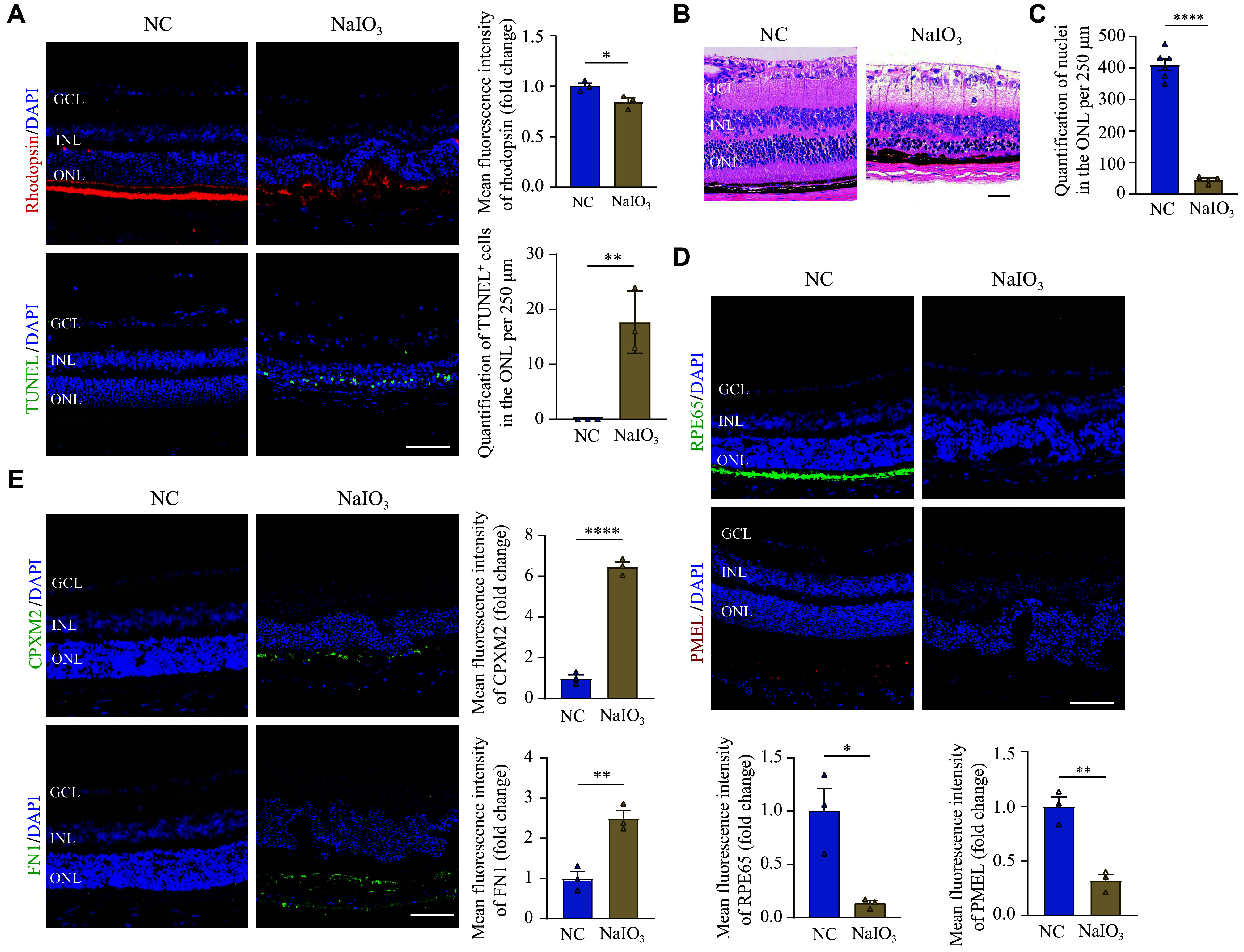
High expression of CPXM2 and EMT-related gene in dysfunctional RPE of atrophic AMD mouse models.

To establish the mouse model of atrophic AMD, we administered tail vein injection of oxidizing chemical NaIO _3_ in mice. NaIO _3_ induces necroptosis of the RPE and photoreceptor cells through oxidative stress-related processes, which is a linked mechanism for the onset and development of atrophic AMD, especially geographic atrophy ^[ 28] ^. The apoptotic signal of TUNEL was intensively stained in rhodopsin-positive photoreceptor cells in the retina of NaIO _3_-induced mice ( ***Fig. 7A***). H&E staining of the mice retina revealed a breaking of RPE monolayer and a significant loss of photoreceptor cells in the outer nuclear layer (ONL) after NaIO _3_ administration ( ***Fig. 7B*** and ***7C***). Additionally, the results of immunofluorescent staining showed that RPE65 (a protein abundantly found in the retinal pigment epithelium, serving as a marker for well-preserved RPE function) and premelanosome protein (PMEL, a key protein for mammalian melanosome biogenesis that plays a pivotal role in the melanosomes maintenance of the RPE and photoreceptor outer segments ^[ 29] ^) were decreased in the retina of NaIO _3_-induced mice, suggesting an RPE dysfunction ( ***Fig. 7D***). These findings confirm the validity of this atrophic AMD mouse model.

Compared with the control mice, RPE cells in the retina of NaIO _3_-induced mice exhibited elevated expression levels of CPXM2 and FN1 ( ***Fig. 7E***). These results verify the oxidative injury and EMT of RPE cells in the atrophic AMD mouse model. Moreover, the increased expression level of CPXM2 also underscores its significance in the pathological process of atrophic AMD.

## Discussion

The latent molecular signals of RPE cells in atrophic AMD could be elucidated by investigating snRNA-seq data and bulk RNA-seq data. Our findings revealed a significant upregulation of oxidative stress and EMT pathways in fibrotic RPE cells associated with atrophic AMD. We identified *CPXM2* as a pivotal gene involved in these pathways. Through further investigations using the oxidative stress-induced cell model and the NaIO _3_-induced mouse model of atrophic AMD, we demonstrated the regulatory role of CPXM2 in RPE cells, revealing a promising target for the atrophic AMD treatment.

Several factors contribute to AMD, including the impairment of RPE cell function, which leads to the progressive degeneration of photoreceptors. A part of the dysfunction of RPE is associated with the transition from mature epithelial cells to mesenchymal cells ^[ 30] ^. This EMT of RPE represents a flexible adaptive process in response to pathological conditions ^[ 31] ^. The remarkable cellular plasticity of the highly specialized RPE cells is underscored by their significant morphological changes. It has been proposed that cells derived from the EMT exhibit an intermediate state, characterized by the varying proportions of epithelial and mesenchymal traits. Cells in this transitional state exhibit a high degree of plasticity and instability, with the ability to partially or completely revert to the original state ^[ 32] ^. Consequently, investigating the regulatory mechanisms governing this mesenchymalization process is crucial for comprehending the progress of atrophic AMD and exploring potential strategies of therapy to counteract the RPE dysfunction.

Orozco *et al* ^[ 20]^ used adequate RPE cells for solid molecular research and built expression atlases of control and AMD donor eyes at both bulk tissues and single-cell levels. In their study, they identified cell type-specific disease characteristics at the single-cell level and molecular changes in each stage of AMD through bulk RNA-seq. However, they did not capture stage-specific cellular signatures, especially for RPE cells. Their work primarily investigated the states of Müller cells at the single-cell level, whereas the current study focused on the factors contributing to the dysfunction of RPE cells in atrophic AMD. Upon analyzing the DEGs derived from the bulk data provided by Orozco *et al*, we observed that the expression pattern of *CPXM2* is consistent with our findings, supporting the correlation between CPXM2 and atrophic AMD. We sought to investigate new mechanisms regulating RPE phenotype by integrating single-cell and bulk RNA-seq data. Additionally, the pathway analysis based on bulk RNA-seq data provides new insights into the pathological microenvironment of atrophic AMD.

*CPXM2* was identified by Orozco *et al* as the most altered gene in fibroblasts ^[ 20] ^. In the current study, *CPXM2* expression in RPE may mediate intermediate plasticization. Orozco *et al* did not elucidate the elevated expression of CPXM2 in fibroblasts of the advanced AMD group. We hypothesize that CPXM2 may be a sensitive indicator of degree-dependent expression of pathological mesenchymal transformation. The fibroblasts exist in a complete mesenchymal state, which makes it unsurprising that their mesenchymal-like level is higher than that of RPE cells with a diminished epithelial phenotype. The consistent reactions of RPE cells and fibroblasts sensing the disease microenvironment may reflect the disease fibrotic response, which indicates the deepithelialization phenotype of RPE in advanced AMD.

CPXM2 belongs to the M14 family and has been reported to be implicated in a poor prognosis and the promotion of tumor aggressiveness in various malignancies ^[ 33] ^. Nonetheless, the role of CPXM2 in the process of RPE depolarization remains poorly understood. As a secretory enzyme, CPXM2 has the potential to activate the transforming growth factor beta (TGF-β) pathway and other routes through the autocrine bypass, akin to the activation of latent TGF-β by matrix metalloproteinases (MMPs) *via* proteolysis ^[ 34] ^. Given these mechanisms, *CPXM2* may conceivably act as a crucial gene involved in the pathological progression of atrophic AMD and may represent a promising therapeutic target.

In conclusion, the current study has shed some light on the underlying processes in RPE cells that may contribute to atrophic AMD, and identified *CPXM2* as a causative gene that participates in the phenotypic transformation of RPE. However, the study also has some limitations, such as the lack of detailed elucidation regarding the specific mechanisms by which CPXM2 promotes the EMT. Nevertheless, the study provides a theoretical basis for the development of novel treatment strategies for atrophic AMD. To deepen our understanding of how CPXM2 influences AMD pathology, future investigation should include additional experimental models.

## SUPPLEMENTARY DATA

Supplementary data to this article can be found online.

## References

[1] (2017). Updates on the epidemiology of age-related macular degeneration. Asia Pac J Ophthalmol (Phila).

[2] (2012). Age-related macular degeneration. Adv Exp Med Biol.

[3] (2016). Histopathological insights into choroidal vascular loss in clinically documented cases of age-related macular degeneration. JAMA Ophthalmol.

[4] (2020). The cell biology of the retinal pigment epithelium. Prog Retin Eye Res.

[5] (2013). Dry age-related macular degeneration: mechanisms, therapeutic targets, and imaging. Invest Ophthalmol Vis Sci.

[6] (2022). Complement cascade inhibition in geographic atrophy: a review. Eye (Lond).

[7] (2023). Avacincaptad pegol: first approval. Drugs.

[8] (2021). Pegcetacoplan: first approval. Drugs.

[9] (2023). Oxidative stress and antioxidants in age-related macular degeneration. Antioxidants (Basel).

[10] (2019). Clinical-grade stem cell-derived retinal pigment epithelium patch rescues retinal degeneration in rodents and pigs. Sci Transl Med.

[11] (2020). EMT and EndMT: emerging roles in age-related macular degeneration. Int J Mol Sci.

[12] (2017). Macular degeneration epidemiology: nature-nurture, lifestyle factors, genetic risk, and gene-environment interactions—the weisenfeld award lecture. Invest Ophthalmol Vis Sci.

[13] (2015). Restoration of mesenchymal retinal pigmented epithelial cells by TGFβ pathway inhibitors: implications for age-related macular degeneration. Genome Med.

[14] (2018). A role for βA3/A1-crystallin in type 2 EMT of RPE cells occurring in dry age-related macular degeneration. Invest Ophthalmol Vis Sci.

[15] (2020). Single-cell RNA sequencing in cardiovascular development, disease and medicine. Nat Rev Cardiol.

[16] (2015). Highly parallel genome-wide expression profiling of individual cells using nanoliter droplets. Cell.

[17] (2017). Single-cell sequencing reveals dissociation-induced gene expression in tissue subpopulations. Nat Methods.

[18] (2019). Advantages of single-nucleus over single-cell RNA sequencing of adult kidney: rare cell types and novel cell states revealed in fibrosis. J Am Soc Nephrol.

[19] (2022). Choroidal endothelial and macrophage gene expression in atrophic and neovascular macular degeneration. Hum Mol Genet.

[20] (2023). A systems biology approach uncovers novel disease mechanisms in age-related macular degeneration. Cell Genom.

[21] (2021). Integrated analysis of multimodal single-cell data. Cell.

[22] (2012). clusterProfiler: an R package for comparing biological themes among gene clusters. OMICS J Integr Biol.

[23] (2021). Sodium iodate-induced degeneration results in local complement changes and inflammatory processes in murine retina. Int J Mol Sci.

[24] (2020). Circular noncoding RNA NR3C1 acts as a miR-382-5p sponge to protect RPE functions
*via* regulating PTEN/AKT/mTOR signaling pathway. Mol Ther.

[25] (2018). Evidence for the activation of pyroptotic and apoptotic pathways in RPE cells associated with NLRP3 inflammasome in the rodent eye. J Neuroinflammation.

[26] (2016). A novel AhR ligand, 2AI, protects the retina from environmental stress. Sci Rep.

[27] (2011). Snail involves in the transforming growth factor β1-mediated epithelial-mesenchymal transition of retinal pigment epithelial cells. PLoS One.

[28] (2016). Retinal pigment epithelial cell necroptosis in response to sodium iodate. Cell Death Discov.

[29] (2015). Regulation of melanosome number, shape and movement in the zebrafish retinal pigment epithelium by OA1 and PMEL. J Cell Sci.

[30] (2020). Role of epithelial-mesenchymal transition in retinal pigment epithelium dysfunction. Front Cell Dev Biol.

[31] (2021). Epithelial-mesenchymal transition and senescence in the retinal pigment epithelium of
*NFE2L2/PGC-1α* double knock-out mice. Int J Mol Sci.

[32] (2016). Emt: 2016. Cell.

[33] (2020). Expression of carboxypeptidase X M14 family member 2 accelerates the progression of hepatocellular carcinoma
*via* regulation of the gp130/JAK2/Stat1 pathway. Cancer Manag Res.

[34] (2016). Regulation of the bioavailability of TGF-β and TGF-β-related proteins. Cold Spring Harb Perspect Biol.

