# Supplementary material for: RNA-sequencing expression profile and functional analysis of retinal pigment epithelium in atrophic age-related macular degeneration
Source: J Biomed Res. 2024 May 29;38(5):500–11. doi: 10.7555/JBR.37.20230320 (PMC11461538; doi:10.7555/JBR.37.20230320)
Supplement: Supplementary file 1 — Supplementary data to this article can be found online. [file jbr-38-5-500-S1.pdf]

Available online at www.jbr-pub.org.cn

Open Access at PubMed Central

Journal of Biomedical Research, 2024 38(5): 1–7

Supplementary Data

JBR

RNA-sequencing expression profile and functional analysis of retinal pigment epithelium in atrophic age-related macular degeneration

Miao Xu△, Yan Gao△, Wenjie Yin, Qinghuai Liu✉, Songtao Yuan✉

Department of Ophthalmology, the First Affiliated Hospital of Nanjing Medical University, Nanjing, 210029, China.

Supplementary Table 1 **Donor statistics**

Datasets	Diagnosis	Eyes (*n*)	Female (*n*)	Male (*n*)	Age (years, mean±SD)	PMI (hours, mean±SD)
Bulk RNA-seq (RPE/choroid macula)	Normal (AREDS 0/1)	8	2	6	77.8±12.3	–
	Geographic atrophy	9	5	4	77.5±12.4	–
Single nucleus RNA-seq (retina+RPE/choroid)	Control	7	2	5	73.3±6.9	4.1±0.7
	AMD	6	4	2	88.2±9.0	5.6±1.1

Abbreviations: PMI, postmortem interval; AREDS, age-related eye disease study; AMD, age-related macular degeneration; SD, standard deviation; –, not counted.

Supplementary Table 2 **Primers used for qRT-PCR**

Genes	Sequences (5′-3′)
CPXM2	Forward: CAGAGGATCGACAGAATGTCCC
	Reverse: CATCCAGGCTATGACTGCTCTG
ACTB	Forward: ACCTTCTACAATGAGCTGCG
	Reverse: CCTGGATAGCAACGTACATGG

△These authors contributed equally to this work.

✉Corresponding authors: Qinghuai Liu and Songtao Yuan, Department of Ophthalmology, the First Affiliated Hospital of Nanjing Medical University, 101 Longmian Avenue, Jiangning District, Nanjing, Jiangsu 211166, China. E-mails: songtaoyuan@njmu.edu.cn (Yuan) and liuqh@njmu.edu.cn (Liu).

Received: 03 January 2024; Revised: 11 March 2024; Accepted: 19 March 2024; Published online: 29 May 2024

CLC number: R774, Document code: A

The authors reported no conflict of interests.

This is an open access article under the Creative Commons Attribution (CC BY 4.0) license, which permits others to distribute, remix, adapt and build upon this work, for commercial use, provided the original work is properly cited.

© 2024 by Journal of Biomedical Research.

https://doi.org/10.7555/JBR.37.20230320

Supplementary Table 3 **Top 50 upregulated and downregulated genes of snRNA-seq in AMD RPE cells**

Genes	*P*_val[a]	log_2(fold change)	pct.1[b]	pct.2[c]	*P*_adj[d]	Regulate
FP236383.3	5.67E-158	1.04	0.74	0.36	3.28E-153	Up
DOCK3	4.65E-146	0.94	0.63	0.27	2.68E-141	Up
AL354733.3	1.97E-121	0.87	0.68	0.36	1.14E-116	Up
FGF12	5.35E-120	1.25	0.59	0.29	3.09E-115	Up
LDLRAD4	9.24E-111	1.02	0.47	0.18	5.33E-106	Up
CUX2	6.82E-108	0.74	0.30	0.07	3.94E-103	Up
L3MBTL4	3.64E-104	0.67	0.35	0.10	2.10E-99	Up
STK39	3.65E-87	0.64	0.93	0.83	2.11E-82	Up
HNRNPK	5.73E-80	0.77	0.84	0.70	3.31E-75	Up
XIST	9.39E-75	0.82	0.55	0.28	5.42E-70	Up
ANKRD37	1.85E-71	0.90	0.37	0.15	1.07E-66	Up
CRYAB	1.87E-68	0.66	0.26	0.08	1.08E-63	Up
SCD	7.88E-62	0.80	0.62	0.39	4.55E-57	Up
CP	1.96E-60	0.59	0.31	0.12	1.13E-55	Up
FP671120.5	2.67E-57	0.62	0.79	0.59	1.54E-52	Up
PTPRZ1	1.11E-55	0.76	0.68	0.45	6.40E-51	Up
LINC01505	9.30E-49	0.72	0.39	0.20	5.37E-44	Up
DMD	2.08E-42	0.64	0.85	0.71	1.20E-37	Up
RASGEF1B	3.53E-42	0.59	0.47	0.29	2.04E-37	Up
HSP90AA1	1.89E-40	0.91	0.75	0.60	1.09E-35	Up
LDLRAD3	1.57E-39	0.74	0.27	0.13	9.07E-35	Up
LINGO1	2.02E-32	0.63	0.55	0.39	1.17E-27	Up
FAM78B	5.64E-30	0.61	0.43	0.27	3.26E-25	Up
PHF10	7.00E-19	0.60	0.43	0.31	4.04E-14	Up
ZNF331	7.63E-11	0.97	0.30	0.22	4.40E-06	Up
AUTS2	4.14E-84	-0.58	0.96	0.99	2.39E-79	Down
STRA6	6.11E-73	-0.57	0.75	0.86	3.53E-68	Down
COL4A3	3.62E-72	-0.51	0.95	0.97	2.09E-67	Down
SLC38A11	3.75E-61	-0.68	0.24	0.48	2.17E-56	Down
CNGB3	4.44E-60	-0.74	0.77	0.86	2.56E-55	Down
PIP5K1B	5.18E-59	-0.54	0.89	0.93	2.99E-54	Down
FLVCR2	4.46E-57	-0.49	0.88	0.95	2.57E-52	Down
RGS7	1.43E-49	-0.63	0.62	0.76	8.27E-45	Down
PRKCQ	1.21E-48	-0.48	0.76	0.84	7.01E-44	Down
APBB2	1.52E-44	-0.52	0.68	0.77	8.78E-40	Down
FREM1	1.80E-43	-0.54	0.35	0.54	1.04E-38	Down
NMNAT3	6.60E-43	-0.52	0.67	0.78	3.81E-38	Down
HUNK	3.18E-42	-0.49	0.27	0.45	1.83E-37	Down
SLC16A14	1.91E-40	-0.52	0.86	0.91	1.10E-35	Down
CAMK1D	2.86E-39	-0.50	0.46	0.63	1.65E-34	Down
AL139220.2	4.51E-38	-0.56	0.47	0.63	2.60E-33	Down
PKHD1	2.07E-36	-0.67	0.19	0.36	1.19E-31	Down
CLDN10	4.07E-33	-0.56	0.44	0.57	2.35E-28	Down
CLDN10-AS1	1.70E-32	-0.50	0.48	0.62	9.79E-28	Down
NELL1	1.44E-31	-0.93	0.06	0.18	8.31E-27	Down
CCDC136	7.13E-29	-0.64	0.53	0.62	4.12E-24	Down
C5orf17	3.44E-26	-0.56	0.05	0.17	1.98E-21	Down
LINC02275	1.27E-21	-0.61	0.42	0.52	7.33E-17	Down
PLAC9	6.94E-16	-0.54	0.96	0.98	4.01E-11	Down
AC092916.1	4.97E-07	−0.66	0.62	0.62	2.87E-02	Down

[a]The original *P*-value obtained after hypothesis testing.
[b]The percentage of cells in the population where the gene expression was detected.
[c]The percentage of cells in other population where the gene expression was detected.
[d]The corrected *P*-value obtained after the Bonferroni multiple test correction.

Supplementary Table 4 **Intersection of differentially expressed genes (DEGs) between pseudobulk analysis from Orozco *et al* and Seurat function in the current study**

Pseudobulk	Seurat	Count	Values
TRUE	TRUE	62	*DOCK3, ESR1, MERTK, GSTO2, CAMK1D, SLC25A42, CD164, HUNK, APBB2, TRAF5, C16orf74, NQO2, APC, STAM, HPS5, SLC38A11, TGFB2, AFDN, ABCA10, SMOC2, ESRRG, CNGB3, L3MBTL4, SHTN1, ATRNL1, COL19A1, PGM5, C1GALT1, FRMD5, SIAH1, KLHL13, SLC4A10, STRA6, GRB10, CDKN1C, AUTS2, LRP1B, STK39, CP, IDI1, RGS7, LDLRAD4, ANKRD37, PFKP, LRP4, FAM13A, CALD1, HNRNPK, MYO1C, ADAM9, ARRDC3, FREM1, FLVCR2, DOK6, SREBF1, LINC00511, SCARB1, MAP3K8, COL11A1, SCD, PTPN14, BOC*
FALSE	TRUE	304	*FP236383.3, AL354733.3, FGF12, CUX2, NEAT1, PVT1, AP002075.1, FOS, XIST, COL4A3, VIM, CRYAB, SPATS2L, PMP22, ANKS1B, GYPA, PIP5K1B, FP671120.5, PTPRZ1, EFR3B, SON, DUSP1, ABR, LAYN, CNTN3, HMOX1, PMEL, LINC01505, PRKCQ, MCTP1, GEM, PLOD2, SLC26A3, NMNAT3, DMD, RASGEF1B, INPP5K, CHI3L1, LURAP1L-AS1, ABCG1, HSP90AA1, SLC16A14, CPXM2, CNTNAP3, LDLRAD3, COL12A1, AL139220.2, UTY, MAN2A1, VEGFA, DDIT4, RLBP1, CTSV, PKHD1, ZBTB16, TPM1, AC012404.1, SIPA1L3, TYRP1, PAXBP1, FRMD4A, ST6GALNAC2, CLDN10, CLDN10-AS1, LINGO1, SLC6A6, ENPP2, RGR, RDH10, LMO1, PITPNA, GNA14, SORBS2, NOVA1, NELL1, ABCA5, PCSK2, FBXO32, UBA6-AS1, KIAA1217, AGBL4, FAM78B, ABHD2, SNAP91, BEST1, ARL17B, VAV3, AC107021.1, CCDC136, HMGCS2, CRYZ, FYB2, DNAH14, SPAG9, DDX5, SGMS1, ADAMTSL1, RAPGEF4, EDIL3, ZC3H7A, C5orf17, ULK1, IQSEC3, GLS, DLG2, SYNE2, BHLHE40, HNRNPDL, GNA14-AS1, PTPRM, PTPRG, RHO, USP9Y, PLCL1, AC116562.3, AC098650.1, MTCL1, FNBP1, HSPA1A, ASAP1, CST3, AC097480.1, ZNF92, NPAS3, RDH10-AS1, WSB1, SNTB1, WWC1, AL139383.1, EYA2, PLA2G5, P4HA1, KIZ, LINC02275, CACNA2D3, MYRIP, NR4A3, GLDC, PAWR, UTRN, XKR6, CDH19, NFIC, ERBIN, RBP1, BASP1-AS1, NCALD, SLC22A6, SUCLA2P3, SLCO1C1, ELOVL5, PRKCQ-AS1, HIF1A-AS3, SLIT3, SPART, WWOX, CHN2, AL139184.1, HSPH1, SEL1L3, HNRNPA2B1, GPC6, FMN1, TSC22D1, SRSF11, PHF10, PTP4A1, SLC12A2, LINC00278, TBC1D2B, RNF150, CMYA5, AC046134.2, SRRM2, CRACD, PTK2B, AC090572.3, CSRP2, XRRA1, SGMS1-AS1, USP53, SMARCA1, CWF19L2, PLEKHA5, TBC1D1, CERS4, NETO2, PCP4, AP003049.2, AC084866.2, SLC23A2, C7orf31, DLG1, AHI1, NELL2, AC019197.1, PLAC9, PAX8-AS1, DST, KSR2, RGS16, RIMS2, LIMD1, SLC6A13, TTTY14, AC022146.2, AFF4, KCNIP4, ABCC5-AS1, PPP4R4, LARP4, ERMN, CPEB3, NLGN1, MYO16, SAT1, ANO10, AL161757.4, ARID5B, SLC30A10, AP1S2, PHLDB2, SPAG16, LINC02307, CGNL1, AC123912.1, WWC2, AC092944.1, TSC22D3, SLC7A5, SLC4A4, HLF, FCHSD2, NCKAP5, CCDC82, NLGN4Y, NXN, CACHD1, SEMA6D, PLXNA2, NABP1, PRMT9, CLYBL, MAML2, DUSP4, IGFBP5, PLCXD3, ERO1A, SNHG25, FHOD3, CNBP, LDLR, TTLL4, LINC02715, SPART-AS1, TMEM184C, KCNJ13, ZNF331, LDB2, AL390783.1, LMCD1-AS1, SAMD4A, PTPRG-AS1, NRG1, IDH1, ADAMTSL4-AS1, INSYN2B, ARG2, GEN1, MITF, GLUD1, ENOX1, ADAMTS19, PKD1L2, VEPH1, ALOX12-AS1, AC010620.2, PLEKHH1, MKX, RYR2, ARL15, PBX3, LARGE1, LRP8, AMPD2, VASN, LAMB1, OSBPL6, ANKRD20A5P, LHFPL6, LINC02343, CCBE1, AC092916.1, KCNQ1OT1, C5orf67, BCO1, CD96, TTC3, PGD, MAT2A, CLSTN2, H3-3B, PDE1C, IGFBP7*
TRUE	FALSE	38	*SLC4A8, PRTG, PPARGC1B, HTRA1, CYFIP2, AF117829.1, PAPSS1, RHOT2, BCO2, ABCA6, HSD17B7, SRC, RHOBTB1, BNIP3L, RETREG3, ZNF133, PAM, CCDC130, MFHAS1, TRIM38, FBN1, HIF1A-AS2, PELI1, C11orf74, FZD3, XKR9, TNS1, MPPED2, PLPP5, CHCHD10, MED12L, HGD, MTUS2, ZCCHC2, LAMC1, NME3, PDE4D, CHST15*

Supplementary Table 5 **Top 50 significantly enriched Gene Ontology (GO) terms of snRNA-seq in AMD RPE cells**

ID	Description	GeneRatio	BgRatio	*P*-value	*Q*-value
GO:0007517	Muscle organ development	18/287	333/18 500	4.94E-06	1.50E-02
GO:0060560	Developmental growth involved in morphogenesis	14/287	234/18 500	1.84E-05	1.69E-02
GO:0060537	Muscle tissue development	19/287	404/18 500	1.95E-05	1.69E-02
GO:0001822	Kidney development	16/287	303/18 500	2.22E-05	1.69E-02
GO:0072001	Renal system development	16/287	312/18 500	3.17E-05	1.93E-02
GO:0001655	Urogenital system development	17/287	352/18 500	3.84E-05	1.95E-02
GO:0034638	Phosphatidylcholine catabolic process	4/287	16/18 500	8.91E-05	3.51E-02
GO:0044089	Positive regulation of cellular component biogenesis	20/287	493/18 500	9.36E-05	3.51E-02
GO:0035725	Sodium ion transmembrane transport	11/287	177/18 500	1.04E-04	3.51E-02
GO:0006814	Sodium ion transport	13/287	249/18 500	1.46E-04	4.44E-02
GO:0007163	Establishment or maintenance of cell polarity	12/287	218/18 500	1.60E-04	4.44E-02
GO:0035335	Peptidyl-tyrosine dephosphorylation	8/287	102/18 500	1.89E-04	4.66E-02

Supplementary Table 5 **Top 50 significantly enriched Gene Ontology (GO) terms of snRNA-seq in AMD RPE cells** (continued)

ID	Description	GeneRatio	BgRatio	*P*-value	*Q*-value
GO:0048545	Response to steroid hormone	15/287	328/18 500	1.99E-04	4.66E-02
GO:0031334	Positive regulation of protein-containing complex assembly	11/287	194/18 500	2.32E-04	4.70E-02
GO:0048588	Developmental cell growth	12/287	227/18 500	2.33E-04	4.70E-02
GO:0060065	Uterus development	4/287	21/18 500	2.76E-04	4.70E-02
GO:0003016	Respiratory system process	5/287	38/18 500	2.86E-04	4.70E-02
GO:0001654	Eye development	16/287	379/18 500	3.02E-04	4.70E-02
GO:0022604	Regulation of cell morphogenesis	14/287	305/18 500	3.11E-04	4.70E-02
GO:0034329	Cell junction assembly	17/287	420/18 500	3.21E-04	4.70E-02
GO:0097006	Regulation of plasma lipoprotein particle levels	7/287	84/18 500	3.29E-04	4.70E-02
GO:0150063	Visual system development	16/287	383/18 500	3.39E-04	4.70E-02
GO:1990138	Neuron projection extension	10/287	172/18 500	3.64E-04	4.81E-02
GO:0048880	Sensory system development	16/287	389/18 500	4.03E-04	4.89E-02
GO:0097105	Presynaptic membrane assembly	3/287	10/18 500	4.09E-04	4.89E-02
GO:0051591	Response to cAMP	7/287	88/18 500	4.37E-04	4.89E-02
GO:0106106	Cold-induced thermogenesis	9/287	146/18 500	4.65E-04	4.89E-02
GO:0120161	Regulation of cold-induced thermogenesis	9/287	146/18 500	4.65E-04	4.89E-02
GO:0040013	Negative regulation of locomotion	16/287	395/18 500	4.76E-04	4.89E-02
GO:2000146	Negative regulation of cell motility	15/287	360/18 500	5.32E-04	4.89E-02
GO:0016049	Cell growth	18/287	480/18 500	5.36E-04	4.89E-02
GO:2000463	Positive regulation of excitatory postsynaptic potential	4/287	25/18 500	5.55E-04	4.89E-02
GO:0097090	Presynaptic membrane organization	3/287	11/18 500	5.56E-04	4.89E-02
GO:0097104	Postsynaptic membrane assembly	3/287	11/18 500	5.56E-04	4.89E-02
GO:0007601	Visual perception	11/287	216/18 500	5.77E-04	4.89E-02
GO:0045834	Positive regulation of lipid metabolic process	9/287	151/18 500	5.94E-04	4.89E-02
GO:1902305	Regulation of sodium ion transmembrane transport	6/287	67/18 500	5.97E-04	4.89E-02
GO:0032273	Positive regulation of protein polymerization	7/287	93/18 500	6.11E-04	4.89E-02
GO:0050953	Sensory perception of light stimulus	11/287	220/18 500	6.72E-04	5.18E-02
GO:0043010	Camera-type eye development	14/287	330/18 500	6.80E-04	5.18E-02
GO:0007585	Respiratory gaseous exchange by respiratory system	6/287	69/18 500	6.99E-04	5.19E-02
GO:0055010	Ventricular cardiac muscle tissue morphogenesis	5/287	47/18 500	7.81E-04	5.67E-02
GO:1990845	Adaptive thermogenesis	9/287	159/18 500	8.60E-04	6.09E-02
GO:0002087	Regulation of respiratory gaseous exchange by nervous system process	3/287	13/18 500	9.42E-04	6.37E-02
GO:0035331	Negative regulation of hippo signaling	3/287	13/18 500	9.42E-04	6.37E-02
GO:0015701	Bicarbonate transport	4/287	29/18 500	9.92E-04	6.38E-02
GO:0030336	Negative regulation of cell migration	14/287	345/18 500	1.04E-03	6.38E-02
GO:0043547	Positive regulation of GTPase activity	12/287	269/18 500	1.06E-03	6.38E-02
GO:0030198	Extracellular matrix organization	13/287	307/18 500	1.07E-03	6.38E-02
GO:0043062	Extracellular structure organization	13/287	308/18 500	1.10E-03	6.38E-02

Supplementary Table 6 **Top 50 upregulated and downregulated genes of bulk RNA-seq from GA samples**

Symbols	log_2FC	log_2CPM[a]	LR[b]	*P*-value[c]	FDR[d]	Regulated
IGHV6-1	6.55	4.60	60.04	9.29E-15	5.79E-11	Up
IGKV2-28	6.39	3.75	82.54	1.03E-19	2.58E-15	Up
IGLV7-43	4.93	6.00	22.13	2.54E-06	2.44E-03	Up
IGHV3-30	4.76	4.12	53.00	3.33E-13	1.66E-09	Up
IGHV3-23	4.44	5.40	60.71	6.62E-15	5.79E-11	Up
IGLL5	4.34	4.15	60.05	9.25E-15	5.79E-11	Up
IGLV1-44	4.22	1.78	40.57	1.90E-10	6.77E-07	Up
IGLV4-69	4.21	3.62	35.90	2.07E-09	5.74E-06	Up
IGKV1-9	4.13	1.72	41.10	1.45E-10	6.02E-07	Up
IL1B	3.35	4.80	27.07	1.96E-07	4.08E-04	Up
IGHV4-61	3.11	0.14	24.95	5.87E-07	8.61E-04	Up
JCHAIN	2.83	7.39	25.69	4.00E-07	6.66E-04	Up
NPVF	2.81	2.24	12.16	4.87E-04	8.20E-02	Up
IGHG4	2.72	2.29	23.15	1.50E-06	1.70E-03	Up
IGLC2	2.69	4.45	36.11	1.86E-09	5.74E-06	Up
TNIP3	2.68	2.69	14.77	1.21E-04	3.88E-02	Up
IGHV3-33	2.65	0.98	26.32	2.89E-07	5.14E-04	Up
IGLV3-10	2.64	0.76	18.72	1.52E-05	9.70E-03	Up
CCL8	2.51	1.55	17.05	3.64E-05	1.68E-02	Up
SELE	2.50	6.30	15.20	9.66E-05	3.30E-02	Up
CD79A	2.41	3.06	26.40	2.78E-07	5.14E-04	Up
RASSF6	2.37	0.77	13.93	1.89E-04	5.12E-02	Up
IL5RA	2.31	0.87	27.43	1.63E-07	3.70E-04	Up
IGHM	2.20	10.62	9.81	1.73E-03	1.47E-01	Up
RND1	2.19	4.86	15.35	8.95E-05	3.12E-02	Up
RASGRF1	−2.86	3.06	9.70	1.84E-03	1.50E-01	Down
CA10	−2.90	2.81	5.24	2.21E-02	3.34E-01	Down
TIGD1	−2.91	2.24	9.06	2.61E-03	1.71E-01	Down
TEC	−2.91	1.32	7.43	6.40E-03	2.39E-01	Down
GABRR1	−2.92	2.16	6.59	1.03E-02	2.69E-01	Down
Lnc-CBLN2-1	−2.96	1.59	6.53	1.06E-02	2.73E-01	Down
OR10AB1P	−2.96	1.83	9.13	2.51E-03	1.66E-01	Down
CDH7	−3.06	2.86	8.33	3.90E-03	2.03E-01	Down
KCNJ6	−3.07	2.47	6.94	8.43E-03	2.56E-01	Down
MIR124-1HG	−3.08	5.58	8.71	3.16E-03	1.85E-01	Down
LOC101929653	−3.09	1.63	6.70	9.62E-03	2.63E-01	Down
GPR179	−3.12	3.79	10.80	1.01E-03	1.13E-01	Down
IGHV1-18	−3.15	4.01	8.82	2.98E-03	1.78E-01	Down
GRM6	−3.15	4.76	11.08	8.72E-04	1.05E-01	Down
GABRA1	−3.22	3.31	7.30	6.89E-03	2.44E-01	Down
ACSL3P1	−3.23	2.34	6.97	8.27E-03	2.53E-01	Down
RN7SL564P	−3.24	−0.12	19.43	1.04E-05	7.23E-03	Down
FUT9	−3.25	1.27	7.30	6.88E-03	2.44E-01	Down
FGG	−3.25	2.01	13.33	2.61E-04	6.13E-02	Down
LRTM2	−3.27	2.32	6.95	8.39E-03	2.55E-01	Down
Lnc-NR2E3-3	−3.32	1.75	10.40	1.26E-03	1.28E-01	Down
IGKV3-11	−3.64	3.08	12.74	3.57E-04	7.06E-02	Down
RN7SL494P	−3.69	0.58	13.66	2.20E-04	5.53E-02	Down
IGHV4-34	−3.78	4.42	10.89	9.67E-04	1.11E-01	Down
OVCH2	−3.82	2.57	9.58	1.96E-03	1.52E-01	Down

[a]Average log_2(Counts per million) across GA and control.
[b]Likelihood ratio statistics.
[c]Unadjusted *P*-value for each gene.
[d]*P*-value adjusted for multiple comparisons.
Abbreviation: GA, atrophic age-related macular degeneration.

Supplementary Table 7 **Top 50 significantly enriched GO terms of bulk RNA-seq from GA samples**

ID	Description	GeneRatio[a]	BgRatio[b]	*P*-value	*Q*-value[c]
GO:0016050	Vesicle organization	142/5 988	304/18 521	1.01E-07	3.64E-04
GO:0043087	Regulation of gtpase activity	165/5 988	364/18 521	1.20E-07	3.64E-04
GO:0007264	Small gtpase mediated signal transduction	215/5 988	499/18 521	2.24E-07	4.53E-04
GO:0010498	Proteasomal protein catabolic process	208/5 988	492/18 521	1.77E-06	2.69E-03
GO:0016482	Cytosolic transport	83/5 988	168/18 521	2.97E-06	3.60E-03
GO:1902115	Regulation of organelle assembly	95/5 988	202/18 521	8.71E-06	6.04E-03
GO:0051056	Regulation of small GTPase mediated signal transduction	132/5 988	298/18 521	9.05E-06	6.04E-03
GO:0051648	Vesicle localization	89/5 988	187/18 521	9.19E-06	6.04E-03
GO:0045494	Photoreceptor cell maintenance	28/5 988	43/18 521	1.03E-05	6.04E-03
GO:0048193	Golgi vesicle transport	129/5 988	291/18 521	1.09E-05	6.04E-03
GO:0051656	Establishment of organelle localization	170/5 988	401/18 521	1.25E-05	6.04E-03
GO:0016236	Macroautophagy	134/5 988	305/18 521	1.28E-05	6.04E-03
GO:0043161	Proteasome-mediated ubiquitin-dependent protein catabolic process	174/5 988	412/18 521	1.29E-05	6.04E-03
GO:0006622	Protein targeting to lysosome	17/5 988	22/18 521	1.95E-05	8.46E-03
GO:0050953	Sensory perception of light stimulus	101/5 988	221/18 521	2.09E-05	8.46E-03
GO:0007601	Visual perception	99/5 988	217/18 521	2.74E-05	1.00E-02
GO:0051650	Establishment of vesicle localization	81/5 988	171/18 521	2.81E-05	1.00E-02
GO:0009583	Detection of light stimulus	38/5 988	67/18 521	3.26E-05	1.10E-02
GO:0007265	Ras protein signal transduction	147/5 988	346/18 521	4.12E-05	1.31E-02
GO:0006887	Exocytosis	150/5 988	355/18 521	4.81E-05	1.46E-02
GO:0016197	Endosomal transport	102/5 988	229/18 521	6.86E-05	1.82E-02
GO:0008654	Phospholipid biosynthetic process	113/5 988	258/18 521	6.88E-05	1.82E-02
GO:0006605	Protein targeting	134/5 988	314/18 521	6.88E-05	1.82E-02
GO:0009584	Detection of visible light	30/5 988	51/18 521	8.69E-05	2.19E-02
GO:0006623	Protein targeting to vacuole	23/5 988	36/18 521	9.96E-05	2.42E-02
GO:0010824	Regulation of centrosome duplication	28/5 988	47/18 521	1.09E-04	2.55E-02
GO:0097731	9+0 Non-motile cilium	70/6 258	132/19 268	7.98E-07	3.26E-04
GO:0097733	Photoreceptor cell cilium	65/6 258	121/19 268	1.07E-06	3.26E-04
GO:0001750	Photoreceptor outer segment	53/6 258	94/19 268	1.47E-06	3.26E-04
GO:0036064	Ciliary basal body	79/6 258	161/19 268	8.53E-06	1.32E-03
GO:0000922	Spindle pole	82/6 258	169/19 268	9.97E-06	1.32E-03
GO:0000151	Ubiquitin ligase complex	132/6 258	301/19 268	2.12E-05	2.35E-03
GO:0005765	Lysosomal membrane	167/6 258	401/19 268	6.25E-05	4.37E-03
GO:0098852	Lytic vacuole membrane	167/6 258	401/19 268	6.25E-05	4.37E-03
GO:0031901	Early endosome membrane	80/6 258	171/19 268	6.44E-05	4.37E-03
GO:0097730	Non-motile cilium	78/6 258	166/19 268	6.58E-05	4.37E-03
GO:0001917	Photoreceptor inner segment	36/6 258	64/19 268	7.37E-05	4.45E-03
GO:0005769	Early endosome	160/6 258	384/19 268	8.54E-05	4.72E-03
GO:0098791	Golgi apparatus subcompartment	159/6 258	383/19 268	1.11E-04	5.68E-03
GO:0030695	GTPase regulator activity	226/6 016	486/18 196	3.19E-10	1.84E-07
GO:0060589	Nucleoside-triphosphatase regulator activity	226/6 016	486/18 196	3.19E-10	1.84E-07
GO:0005096	GTPase activator activity	131/6 016	272/18 196	1.45E-07	5.57E-05
GO:0005525	GTP binding	168/6 016	375/18 196	1.17E-06	2.86E-04
GO:0019001	Guanyl nucleotide binding	176/6 016	397/18 196	1.49E-06	2.86E-04
GO:0032561	Guanyl ribonucleotide binding	176/6 016	397/18 196	1.49E-06	2.86E-04
GO:0005085	Guanyl-nucleotide exchange factor activity	107/6 016	223/18 196	2.42E-06	3.97E-04
GO:0003924	GTPase activity	146/6 016	332/18 196	1.88E-05	2.70E-03
GO:0031267	Small gtpase binding	119/6 016	267/18 196	5.27E-05	6.74E-03
GO:0016877	Ligase activity, forming carbon-sulfur bonds	26/6 016	41/18 196	6.54E-05	7.53E-03
GO:0070273	Phosphatidylinositol-4-phosphate binding	20/6 016	29/18 196	8.16E-05	8.54E-03

[a]The ratio of the number of genes enriched from the gene list to the target pathway to the total gene set contained in the gene list.
[b]Proportion of target pathway genes to total pathway genes.
[c]*P*-value adjusted for multiple comparisons.
Abbreviations: GO, Gene Ontology; GA, atrophic age-related macular degeneration.

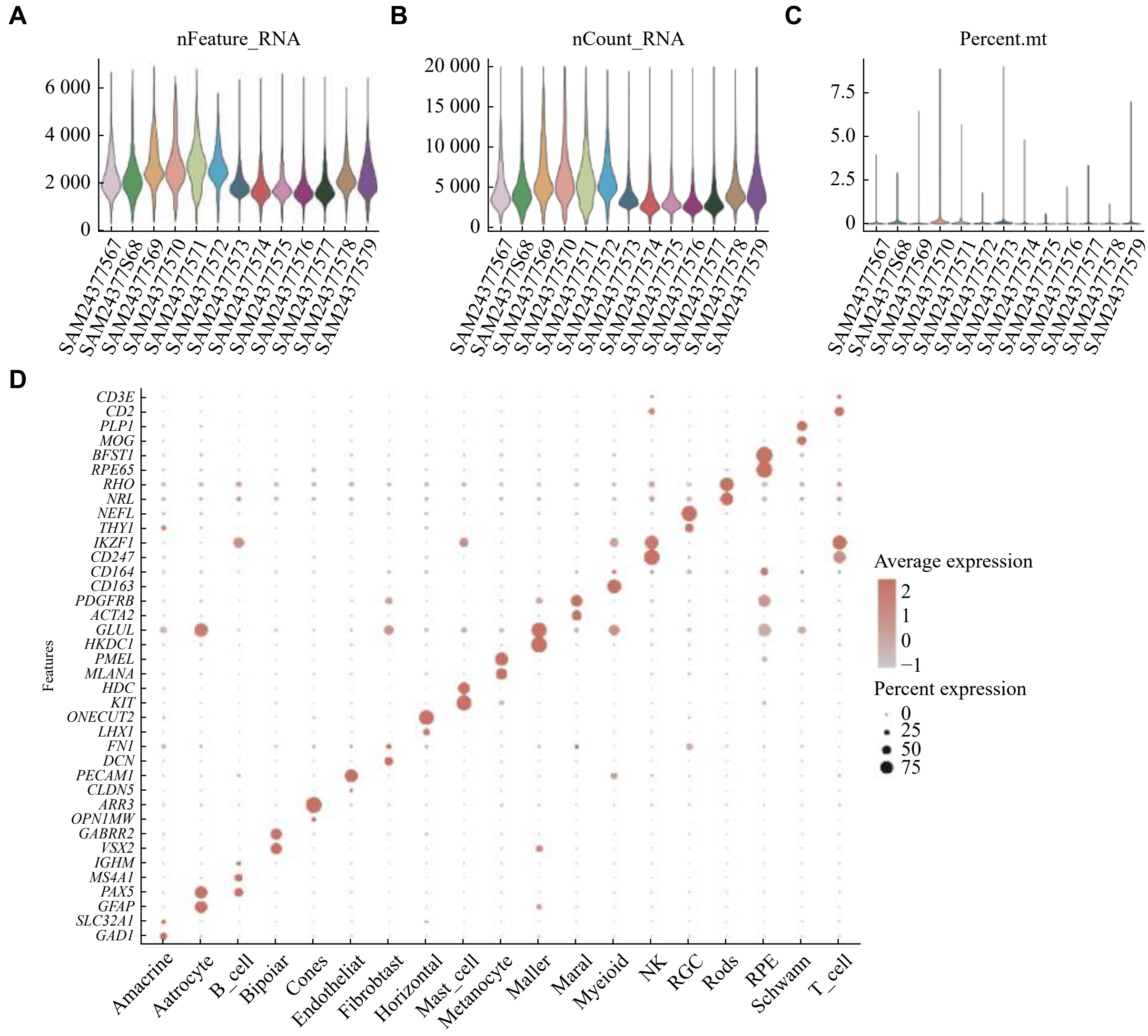

Supplementary Fig. 1 **Quality control of snRNA-seq.** A–C: snRNA-seq of different samples showed the distribution of gene number (nFeature_RNA), molecule number (nCount_RNA), mitochondrial content (percent.mt) in cells. D: Marker gene expression according to the 19 cell types.
